# The Application of Essential Oil Vapors at the End of Vacuum Cooling of Fresh Culinary Herbs Promotes Aromatic Recovery

**DOI:** 10.3390/foods10030498

**Published:** 2021-02-26

**Authors:** Ginés Benito Martínez-Hernández, Amaury Taboada-Rodríguez, Alberto Garre, Fulgencio Marín-Iniesta, Antonio López-Gómez

**Affiliations:** 1 Food Safety and Refrigeration Engineering Group, Department of Agricultural Engineering, Universidad Politécnica de Cartagena, Paseo Alfonso XIII 48, 30203 Cartagena, Spain; 2 Group of Research Food Biotechnology-BTA, Department of Food Science, Nutrition and Bromatology, Campus de Espinardo, University of Murcia, 30100 Murcia, Spain; ataboada@um.es (A.T.-R.); fmarin@um.es (F.M.-I.); 3 Food Microbiology Group, Wageningen University & Research, P.O. Box 17, 6700 AA Wageningen, The Netherlands; alberto.garreperez@wur.nl; 4 Biotechnological Processes Technology and Engineering Lab, Instituto de Biotecnología Vegetal, Edif I+D+I, Campus Muralla del Mar, Universidad Politécnica de Cartagena, 30202 Cartagena, Spain

**Keywords:** vapor EOs, VOCs, SPME, aromatic herbs, parsley, basil, dill, quality, vacuum cooling

## Abstract

Aroma is an important quality parameter of fresh culinary herbs that may be highly affected after postharvest treatments. The innovative technology of vapor essential oil (EO) application under vacuum conditions may recover aroma lost during the postharvest processing of plant products like aromatic herbs. Hence, this study assessed the aroma recovery effect of vapor EOs applied during vacuum cooling on curly parsley and dill. The volatile organic compounds (VOCs) profiles of these aromatic herbs were studied by static headspace solid-phase microextraction (SPME), and the VOCs sorption kinetics onto the SPME stir-bar coating were modeled by the Baranyi model. At the pilot plant scale, the total VOCs contents of parsley and dill (whose extractability was increased by 10–20% after a single vacuum process) were enhanced by 4.5- and 2-fold, respectively, when vapor EOs were applied. In particular, 1,3,8-p-menthatriene and carvone (parsley) increased by 18.7- and 7.3-fold, respectively, while dill ether (the characteristic VOC of dill) augmented by 2.4-fold after vapor EOs were applied under vacuum conditions. The aroma recovery of culinary herbs was successfully validated at an industrial level in an installation developed by our group to apply vapor EOs within a vacuum cooling system, reaching total VOC recoveries of 4.9- and 2.3-fold in parsley and dill, respectively.

## 1. Introduction

Humanity has used aromatic herbs since ancient times for culinary operations to enhance food taste, alongside their well-known high contents of health-promoting compounds [1,2]. As the name indicates, the aroma of these herbs is a crucial quality parameter that plays an important role in the consumer purchase decision. Nevertheless, metabolic processes during the postharvest life of these plant products lead to product quality loss [3]. In this sense, postharvest techniques are needed to extend the product shelf life [4]. Conventional sanitizing washing treatments (e.g., NaOCl) must be avoided in aromatic herbs since these physical operations (washing, rinsing and drying) may highly damage the plant organ, leading to accelerated senescence. Hence, innovative postharvest techniques that do not induce damages to sensitive products like aromatic herbs, while still meeting the consumer interests, are needed: natural food products free from additives obtained with environmental-friendly processing conditions.

Essential oils (EOs) are oily liquids extracted from plants that are widely studied for their high antimicrobial activity. Furthermore, EOs are classified as “Generally Recognized As Safe” (GRAS) by the American Food and Drug Administration (FDA) and allowed as flavorings within the European Union [5,6]. In particular, carvacrol is the major component of oregano EOs with wide spectra against both gram-negative (e.g., enterobacteria) and gram-positive bacteria, and other microbial groups like molds [7,8]. EOs are also considered as strong antioxidants and are likewise able to inhibit the activity of enzymes that degrade product quality, like polyphenol oxidase, and of enzymes involved in the ethylene biosynthesis pathway [9,10]. Thereby, in previous scientific studies from group, we studied that active packaging with EOs can extend the product shelf life by reducing product weight loss, softening, color loss, etc. in several products like culinary herbs, tomatoes, lettuce, peppers, mandarins and grapes [11,12,13].

The use of the “surface decontamination of solid food with vapor EOs under vacuum conditions” (SDuVC) technology developed by our group is an innovative approach to apply EOs in the vapor phase to food products [14]. It is based on the principle that EO vaporization is more effective to treat food products at the vacuum levels of the vacuum cooling systems (about 5–6 hPa) than at atmospheric pressure (about 1000 hPa) [1]. Thus, the treatment agent (EOs) reaches the product surface more homogeneously in its vapor phase, highly increasing the treatment’s effectiveness. The adaption of this technology to the vacuum cooling technique (commonly used to precool produce, mainly leafy vegetables) is an innovative concept in the postharvest field combining the application of EOs in the vapor phase within vacuum cooling installations [1]. Nevertheless, vacuum conditions might lead to the loss of aroma quality of aromatic herbs, as it has been studied in liquid food products (fruit juices and milk) and bread [15,16,17]. However, to the best of our knowledge, the aroma profile response of produce under vacuum conditions to vapor EOs has not been deeply studied.

The aroma profile of aromatic herbs, mainly due to biogenic volatile organic compounds (VOCs), has been widely studied using conventional sample preparation methods for the evaluation of plant emissions, which involve destructive and time-consuming approaches, such as solvent extraction and/or distillation, and are prone to artefacts formation [18]. Some of these artefacts include chemical changes of monoterpenes under conditions of steam distillation and losses of the most volatile compounds during the step of solvent removal of conventional solvent extractions [19]. The static headspace solid-phase microextraction (hereinafter SPME) is a technique that was introduced and later developed by Pawliszyn and co-workers [20,21]. The SPME technique is a fast, solvent-free, automatable and inexpensive method to analyze the aroma profile of food products without changes in their natural flavor pattern, giving a more realistic aroma profile similar to that perceived by the consumer. This technique concentrates the aroma volatiles onto a fiber coated with a thin film of sorbent/adsorbent, which is exposed to the headspace of the plant sample until it reaches equilibrium related to: (i) the plant/headspace equilibrium and (ii) the headspace/fiber accumulation equilibrium [18]. Although reaching the equilibrium is of the high importance for the SPME technique, such a sorption process has not been conveniently modeled for culinary herbs’ VOCs. Finally, volatiles from the SPME fiber are desorbed, usually by heat, into the same gas chromatograph where quantification is done.

This study aimed to evaluate the effect of the vapor EO treatment (carvacrol:spearmint EO mix) on the volatile profile of aromatic herbs (curly parsley and dill) to evaluate a possible recovery of the typical aroma profile of aromatic herbs after vacuum cooling.

## 2. Materials and Methods

### 2.1. Materials

Curly parsley (*Petroselinum crispum* ssp. *crispum* L.) and dill (*Anethum graveolens* L.) were obtained from the company Agroherni SCL (Las Palas, Región de Murcia, Spain) in May 2019. These aromatic herbs were selected on the basis of their economic importance as culinary herbs and their high postharvest metabolic rates (high respiration rate and ethylene production) [3]. These herbs were grown in the Southeast of Spain under greenhouse conditions according to integrated pest management cultural practices. The edible part (stem and leaves) of curly parsley at the mature stage (leaf stems with at least three segments) was hand-harvested, cutting no more than one-third of the growing plant. The edible part (stem and leaves) of dill was also hand-harvested as soon as the plant had four to five leaves. In any case, the plants were always harvested before the plant blooming. Harvested plant material was packaged with crushed ice (except for basil to avoid chilling injury to this very chill-sensitive herb) and then transported ≈30 km to the pilot plant of the Food Safety and Refrigeration Engineering Group (Universidad Politécnica de Cartagena, Spain). The aromatic herbs were stored at 8 °C and 90–95% relative humidity (RH) until the next day when they were used. Carvacrol and spearmint EOs (composition detailed in Table 1) were obtained from Lluch Essence SL (Barcelona, Spain).

SPME stir bars (10 mm long) covered with a 0.5 mm film of polydimethylsiloxane (‘Twisters^®^/Stir Bar Sorptive Extraction; Gerstel, Mülheim an der Ruhr, Germany) were used for SPME determination of VOCs. Before use, SPME stir bars were reconditioned by heating them in the hot injection port of a gas chromatograph–mass spectrometer (GC–MS) at 300 °C for 45 min to remove contaminants.

### 2.2. Volatiles Analysis by Headspace Solid-Phase Microextraction (SPME)

**Figure 1:**
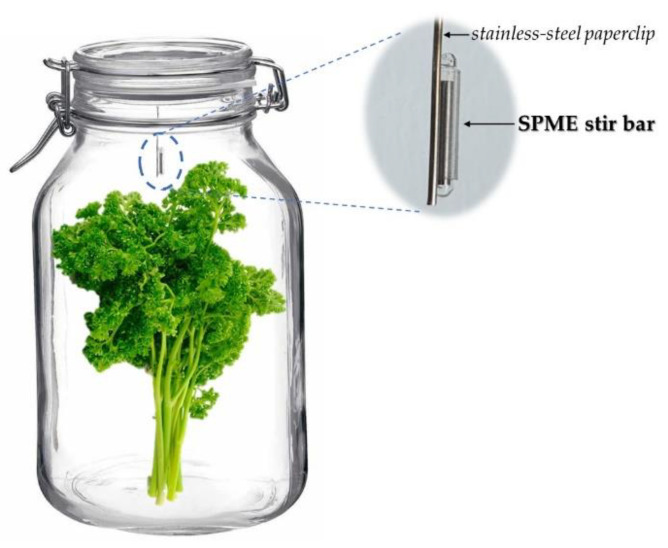
Sampling system for volatile organic compounds (VOCs) of fresh aromatic herbs with static headspace solid-phase microextraction (SPME).

The sampling system consisted of herbs (30 g) placed inside a 3 L airtight glass jar (type Mason jars) with hinged lid and airtight rubber seal (Figure 1). Then, a conditioned SPME stir bar was suspended from the inner side of the lid using a stainless-steel paperclip (see detail from Figure 1), and the lid was immediately closed to start VOCs sorption at room temperature until equilibrium (sorption equilibrium between the analyte and the SPME stir-bar coating) was reached (see Section 2.3). The sorption time was assayed up to 3 h. Finally, the SPME stir bars were removed, gently rinsed with distilled water and carefully dried with laboratory filter paper before the GC–MS analysis.

The analysis of VOCs adsorbed onto the stir-bar coating was done in a GC–MS HP-6890N coupled to a 5975 mass spectrometer (Agilent Technologies, Palo Alto, USA) combined with a TDU and cooling injector system (CIS4) (Gerstel, Mülheim an der Ruhr, Germany), as previously described [22]. Briefly, heat desorption was done from 40 to 250 °C at 100 °C min^−1^ with a 5 min hold time on splitless mode. A cool trap at −100 °C was used to capture the desorbed compounds. VOCs separation was done using an HP5MS-UI column (Agilent Technologies, Palo Alto, CA, USA) with helium as the gas carrier in constant pressure mode, and a split ratio of 1:50. The initial temperature was 50 °C, increasing until 70 °C at a ratio of 5 °C min^−1^, and then held for 1 min. In the next step, the temperature was increased until 240 °C at 10 °C min^−1^, and then held for 15 min. The mass spectrometer operated at 70 eV ionization voltage. The source and quadrupole temperatures were 230 and 150 °C, respectively. The mass range was 30.0 to 450.0 uma at 4 scan s^−1^. The MSD transfer line was maintained at 280 °C. The ChemStation software (version E.02.02 SP1, Agilent Technologies, Palo Alto, CA, USA) was used to acquire chromatograms and peak areas. VOCs were qualitatively identified by comparison with the mass spectral database Willey10th-NIST11b (Agilent Technologies, Wilmington, CA, USA), considering match qualities above 90%. The aroma profile of herbs was also presented as relative (%) abundance. Quantification of the differences among the studied treatments was done using the obtained VOCs peak areas [18,23].

### 2.3. Model of SPME Sorption of Volatiles from Aromatic Herbs

Basil (*Ocimum basilicum* L.) was used as a model aromatic herb to study the SPME sorption of VOCs since this herb has a complex VOCs profile. Basil was grown under the same conditions by the company detailed in Section 2.1.

The VOCs sorption was modeled using the Baranyi model [24]. Although this model was initially suggested to describe the growth of microbial populations, nowadays it is commonly used in a large variety of fields to model growth curves with sigmoidal shapes. We used the algebraic solution calculated by Buzrul and Öksüz [25], shown in Equation (1), where *C* is the VOC peak area, *t* is the sorption time (min), *log* refers to the decimal logarithm and *ln* to the natural logarithm.

(1) $$logC=logC_{max}+log\left(\frac{1+exp\left(ln\left(10\right)\cdot \mu \cdot \left(t-\lambda \right)\right)-exp\left(-ln\left(10\right)\cdot \mu \cdot \lambda \right)}{exp\left(ln\left(10\right)\cdot \mu \cdot \left(t-\lambda \right)\right)-exp\left(-ln\left(10\right)\cdot \mu \cdot \lambda \right)+10^{logC_{max}-logC_{0}}}\right)$$

This model has four parameters that describe the shape of the sigmoidal growth curve. *C_0_* stands for the initial peak area and *C_max_* for the maximum peak area observed during the stationary phase. The duration of the lag phase is described by the parameter *λ* (min), whereas the slope of the curve during the exponential adsorption phase is given by the specific adsorption rate, *µ* (1/min).

The values of the parameters from Equation (1) were estimated independently for each compound using the “biogrowth” package [26] for R version 3.6.3 [27]. We used the function “fit_isothermal_growth”, which fits this adsorption model using non-linear regression. The goodness of the fit was evaluated by a visual inspection of the residuals and quantified using the root-mean-squared error (RMSE). The R code used for the model fitting is available upon request from the corresponding authors of the manuscript.

### 2.4. Effect of Vapor EOs Applied under Vacuum on Volatiles of Aromatic Herbs at Pilot Plant Scale

**Figure 2:**
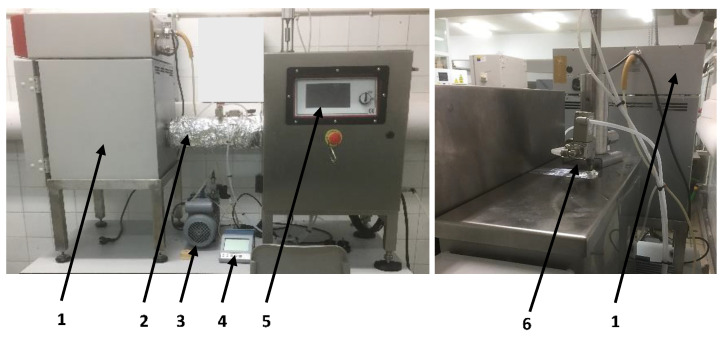
EO vaporization device at the pilot plant scale based on the “surface decontamination of solid food with vapor EOs under vacuum conditions” (SDuVC) technology. The numbers refer to: (1) vacuum camera, (2) isolated tube, (3) vacuum pump, (4) vacuum control system, (5) control panel and (6) evaporator unit. (Reprinted from “Fresh culinary herbs decontamination with essential oil vapors applied under vacuum conditions”, 156, 110942, López-Gómez et al., Copyright (2019), with permission from Elsevier.).

The evaluation of aroma recovery of aromatic herbs with vapor EOs at the pilot plant scale was done using our developed EO vacuum-vaporization pilot plant as previously described [1], and shown in Figure 2. Briefly, the installation was composed of three different interconnected units: a vacuum camera (internal volume of 22.7 L) coupled to a vapor generator and controlled by a control panel. Aromatic herbs were placed over a tray inside the vacuum camera. EO vaporization occurred at 50 °C and it was automatically applied under a 6–8 hPa vacuum. The application of EO vapors was performed in this pilot plant device for 8 min in intermittent vaporization periods of 40 s followed by 20 s of non-vaporization. An EO mix of carvacrol:spearmint EOs (80:20 *v/v*) at 1.2 mg L^−1^ (mg of EOs per liter of the vacuum camera) was used on the basis of our preliminary experiments related to its high antimicrobial effect and quality preservation of the treated herbs [1]. Furthermore, vacuum treatment without EO application (single vacuum) was done to study the effect of the vacuum process itself. Plant materials without any treatment (vacuum or EO) were used as control (CTRL). After the treatments, the VOCs analyses by SPME were immediately done as described in Section 2.2.

### 2.5. Validation at Industrial Scale of Aroma Recovery with Vapor EO Treatment of Aromatic Herbs

**Figure 3:**
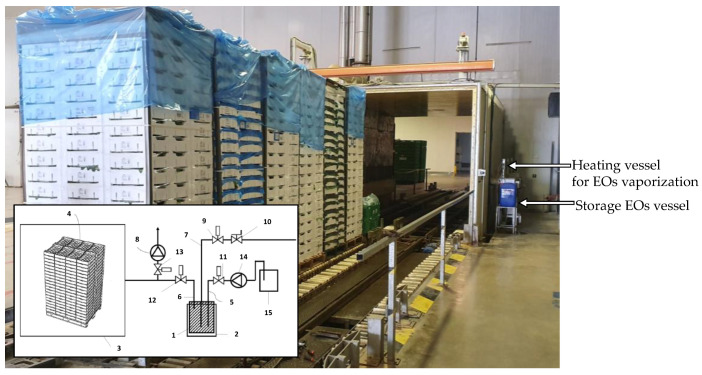
Industrial vacuum cooling system coupled to an essential oil (EO) vaporization installation, based on the SDuVC technology. In the schema of the bottom left-corner: (1) is the EOs evaporator heated by (2) a jacket; (3) is the vacuum camera where EO vapors are applied (at the end of the vacuum cooling process) on (4) the fresh produce packaged in open cases; (5), (6) and (7) are connection tubes and (8) is a vacuum pump; (9), (10), (11), (12) and (13) are valves; and liquid EOs are dosed with (14) a dosing pump, taking the EOs from (15) a jerrycan.

The experimental validation of the vapor EO mix (carvacrol:spearmint EOs, 80:20 *v/v*) treatment under vacuum conditions was done at the industrial scale using a vacuum cooling system (73 m^3^, Figure 3) adapted by our group for the SDuVC technology, similar to that previously described [1]. Previously, aromatic herbs were distributed in boxes (60 × 40 × 22 cm size, expanded polystyrene), which were then disposed of inside this installation. EO vaporization occurred at 50 °C. Then, EOs were automatically applied under a 6–8 hPa vacuum at the end of the vacuum cooling process. The EO vapor treatment time was 8 min. Plant material without vacuum and EO treatment was used as a control (CTRL). Finally, VOCs analyses by SPME were immediately done, as described in Section 2.2.

### 2.6. Statistical Analyses

Statistical analysis was performed using SPSS software (v.19 IBM, New York, NY, USA). Pairwise comparison was done using an independent samples *t*-test. Multiple groups were compared using analysis of variance (ANOVA), followed by Tukey’s Honest Significant Difference post hoc test. In every case, statistical significance was assessed at *p* = 0.05.

## 3. Results and Discussion

### 3.1. SPME Sorption of Volatiles from Aromatic Herbs: Basil as a Model Herb

Following the SPME principle (sorption equilibrium between the analyte and the SPME stir-bar coating), this technique requires careful optimization to obtain high sensitivity and a good repeatability of determination [19]. Thus, extraction time (when equilibrium is reached) influences the VOCs sorption onto the SPME stir-bar coating, which is highly correlated with the extraction temperature. Extraction times are reduced when the SPME extraction temperature is increased [23], with optimum SPME extraction temperatures ranging from 40 to 60 °C for basil and other aromatic herbs [23,28,29]. Nevertheless, high extraction temperatures may involve losses of the most volatile compounds and the risk of chemical changes of some analytes (e.g., monoterpenes) [19]. In accordance, Klimánková et al. [19] recommended an SPME extraction temperature lower than the one determined as the optimum temperature (40 °C), after studying five different basil cultivars, to avoid possible losses of most volatile compounds and volatile chemical changes. Furthermore, the study of most volatile compounds was considered crucial in this experiment since such VOCs highly influence the consumer purchase decision related to the perceived aroma of aromatic herbs. Hence, we chose an SPME extraction temperature of 22 °C (room temperature) in our study.

**Figure 4:**
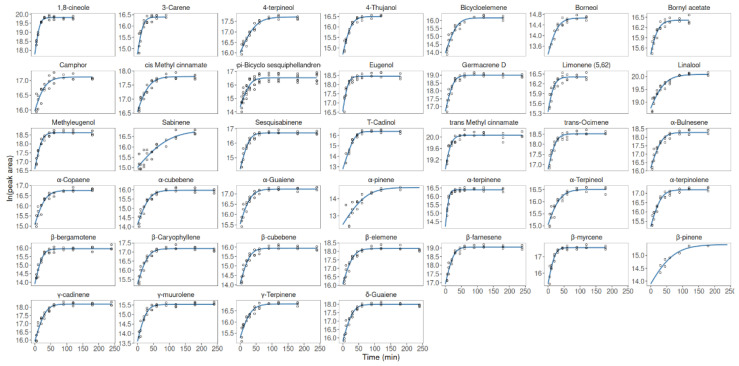
Volatile organic compounds sorption of fresh basil by SPME. Experimental data (symbols) were fitted (lines) with the Baranyi model (described by Equation (1)).

As observed in Figure 4 and Table 2, the Baranyi model was able to describe the temporal variation of the data, with the RMSE (ln peak area) within 0.02–0.04. As expected, VOCs sorption did not show a lag phase (Figure 4), and consequently, we fixed parameter *λ* = 0 min (i.e., no lag phase) before fitting the model with the remaining parameters. Finally, after the stationary phase, the VOCs showed a decay phase (after 2–3 h of sorption), which was omitted for model fitting since such decay type is not described by the Baranyi model.

Among the VOCs, α-terpinene showed the highest affinity for the used SPME stir-bar coating with a sorption rate of 0.042 min^−1^, followed by 1,8-cineole with 0.0296 min^−1^ (Table 2). Eugenol, *β*-myrcene and 3-carene presented intermediate affinity values with sorption rates of 0.0260, 0.0223 and 0.0229 min^−1^, respectively. In general, the oxygenated monoterpenes group (except for 1,8-cineole) had the lowest sorption rates of <0.015 min^−1^, followed by the sesquiterpene hydrocarbons group with sorption rates of 0.012–0.18 min^−1^. Among the SPME coatings, polydimethylsiloxane (which operates in sorption mode) has been successfully used with higher responses in fresh aromatic herbs than other coatings like polyacrylate or mixed phases (which operate both in sorption and adsorption mode), like polydimethylsiloxane/divinyl-benzene, carbowax/divinylbenzene and polydimethylsiloxane/carboxen [30].

**Figure 5:**
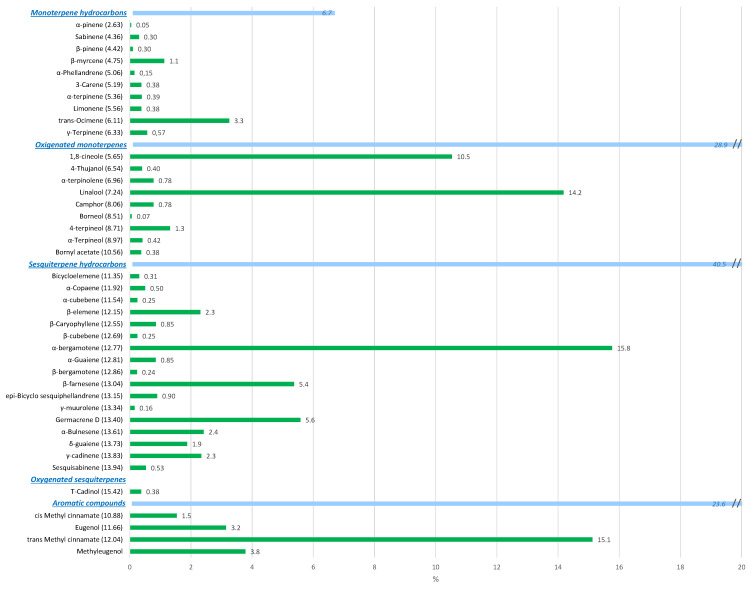
Volatile organic compounds (VOCs) profile of basil expressed as relative percentages. Bars represent relative VOCs contents (%). Values in parentheses represent VOCs retention times.

Among basil VOCs groups, sesquiterpene hydrocarbons represented the major group with 40.5% of the total VOCs content, while oxygenated monoterpenes and aromatic compounds reached 28.9% and 23.6%, respectively (Figure 5). In particular, *α*-bergamotene, trans-methyl cinnamate, linalool and 1,8-cineole were the major identified VOCs with 15.8%, 15.1%, 14.2% and 10.5%, respectively. Most of basil’s VOCs contribute to its aroma background, which is characterized by fragrant, sweet and fresh nuances [31]. In particular, bergamotene is characterized by a fragrant, sweet and fresh aroma. Linalool also adds a woody character to basil [31]. Interestingly, 1,8-cineole is one of the basil VOCs with a more complex and nuanced aroma profile, with fragrant, sweet, cooling, fresh, slightly green and minty nuances [31]. Trans-methyl cinnamate is also a complex aroma, with notes that are sweet, balsamic and reminiscent of cinnamon [32]. Finally, the intermediate contents (≈2–5%) of *β*-elemene and trans-8-farnesene add the green nuances, while a slightly sour character is added by germacrene D (5.6%) [31].

The basil genus (*Ocimum*) has a long list of subspecies and varieties due to its abundant cross-pollination, which makes the taxonomy of the group difficult, and it is classified into three large groups: European type, Exotic or Reunion type and African type [33]. Basil plants possessing a single biosynthetic pathway were classified early by Lawrencet [34] into five chemotypes (depending on the major component: methyl chavicol, methyl eugenol, trans-methyl cinnamate, eugenol or linalool) in a study with oils of more than 200 individual basil plants grown in Eastern North Carolina. Nevertheless, two distinctly different biosynthetic pathways can exist in basil plants, unlike many other *Lamiaceae*, depending upon the genome growth. In another study, the EOs of 12 different basil varieties grown in Colombia exhibited methyl cinnamate, which belongs to the shikimate pathway (which produces aromatic compounds and their derivatives), as one of the most abundant components, in agreement with our study. The considerable amounts of 1,8-cineol and linalool in our study, both from the mevalonic acid pathway (which produces only monoterpenes), indicate the dual biosynthetic pathway of this basil variety. Similarly, Viña and Murillo [33] reported the basil subtype methyl cinnamate > linalool > 1,8-cineole.

### 3.2. Volatiles Profiles of Aromatic Herbs

A comparison of the VOCs profiles of aromatic herbs with previous literature should be done with special attention since, depending on the SPME sorption parameters (mainly temperature and time) and type of SPME coating (since volatile compounds may have different SPME-affinities depending on the SPME coating), different profiles may be obtained. In addition, higher differences may be found between the SPME technique (more volatile compounds) compared with conventional extraction techniques of EOs like steam distillation; a more complete volatile profile is obtained, although most volatile compounds are lost.

#### 3.2.1. Curly Parsley

**Figure 6:**
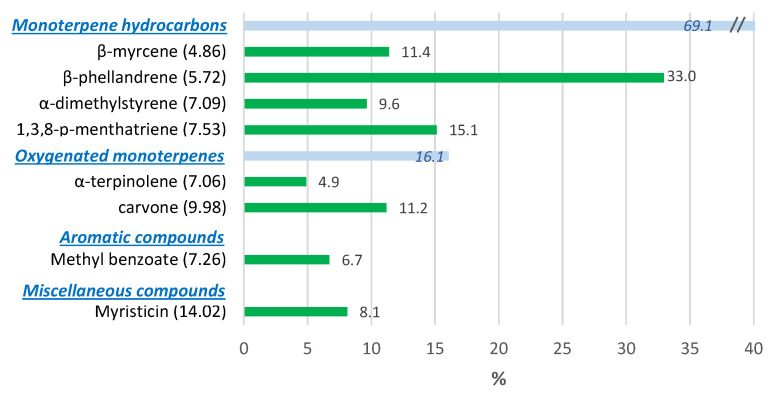
Volatile organic compounds (VOCs) profile of curly parsley expressed as relative percentages. Bars represent relative VOCs contents (%). Values in parentheses represent VOCs retention times.

The VOCs profile of curly parsley by SPME is presented in Figure 6. Monoterpene hydrocarbons were the major group, with *β*-phellandrene as the major component with 33.0% from the total volatiles, followed by 1,3,8-p-menthatriene and *β*-myrcene with 15.1% and 11.4%, respectively. The oxygenated monoterpenes α-terpinolene and carvone accounted for 4.9% and 11.2%, respectively, while myristicin and methyl benzoate showed levels of 8.1% and 6.7%, respectively. Our results agree with previous data that identified *β*-phellandrene, 1,3,8-p-menthatriene, myristicin and myrcene as the major components of the EOs from the aerial parts of more than 100 accessions of parsley [35,36]. Although there is a high variability of volatiles depending on the parsley accession chosen, *β*-phellandrene, 1,3,8-p-menthatriene, α-dimethylstyrene and terpinolene, as well as myristicin and myrcene, are considered to be responsible for the characteristic aroma of parsley [35,37,38,39].

Parsley comprises volatiles derived from mevalonic acid and aromatic polypropanoids biosynthetic pathways [40]. In accordance with our VOCs profile, a similar chemotype was previously reported in Saudi Arabia’s parsley by SPME with *β*-phellandrene as the major volatile (32%) [40]. Nevertheless, myristicin was found as the major volatile (≈30%) in the EO of curly parsley obtained by hydrodistillation [38]. Furthermore, high quantitative variations were obtained when comparing hydrodistillation with SPME in parsley, which is likely because of the key differences in these extraction techniques [40]. In particular, hydrodistillation may induce possible transformations of aroma-active compounds because of heat, steam and pH, leading to losses, the degradation of some volatile compounds due to long extraction times and the degradation of unsaturated or ester compounds through thermal or hydrolytic effects (compiled by Farouk et al. [40]). Then, SPME was proposed for studying the volatile chemical constituents of aromatic plants like parsley without EO extraction [40], giving more realistic data of parsley sensory aroma to differentiate among different samples.

#### 3.2.2. Dill

**Figure 7:**
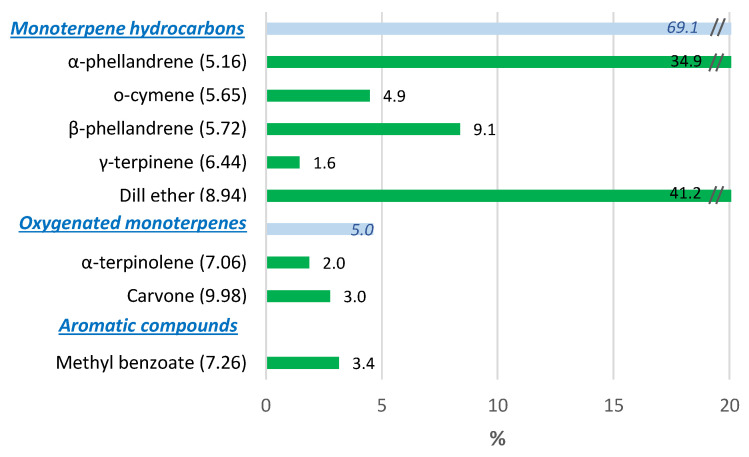
Volatile organic compounds (VOCs) profile of dill expressed as relative percentages. Bars represent relative VOCs contents (%). Values in parentheses represent VOCs retention times.

As observed in Figure 7, dill ether was the major volatile (41.2%) in dill followed by α-phellandrene (34.9%). Minor (2–9%) identified volatiles were *β*-phellandrene, o-cymene, methyl benzoate, carvone, *α*-terpinolene and *γ*-terpinene. Dill ether was previously assessed as the character impact compound of dill herb flavor [41,42], which is described as herbal, dill and spicy [43]. Meanwhile, α-phellandrene adds citrus, herbal, terpene, green, woody and peppery nuances [43]. Dill ether and α-phellandrene were also the major volatiles identified in dill by SPME [44]. Those authors also reported that dill stems had a higher dill ether content, but lower α-phellandrene content, than dill leaves.

Dill ether and α-phellandrene, together with myristicin and methyl 2-methylbutanoate, were established as the compounds that form the important aroma nuance of fresh dill by GC–olfactometry and odor activity value (ratio of concentration to odor threshold) [42]. This also agreed with the ranking of the important odorants in dill published previously by Huopalahti [41]. Although limonene came next to myristicin in Huopalahti’s ranking, where it is reported as a minor (3–9%) VOC in dill [42,43,44,45], it has been considered of minor importance for the overall flavor of dill [42]. The absence of these minor VOCs in our study may be mainly attributed to differences with the literature related to the dill accessions and analytical procedures used (hydrodistillation, SPME, etc.).

### 3.3. Enhancement of VOCs Biosynthesis after Vapor Essential Oils Applied under Vacuum Conditions at Pilot Plant Scale

**Figure 8:**
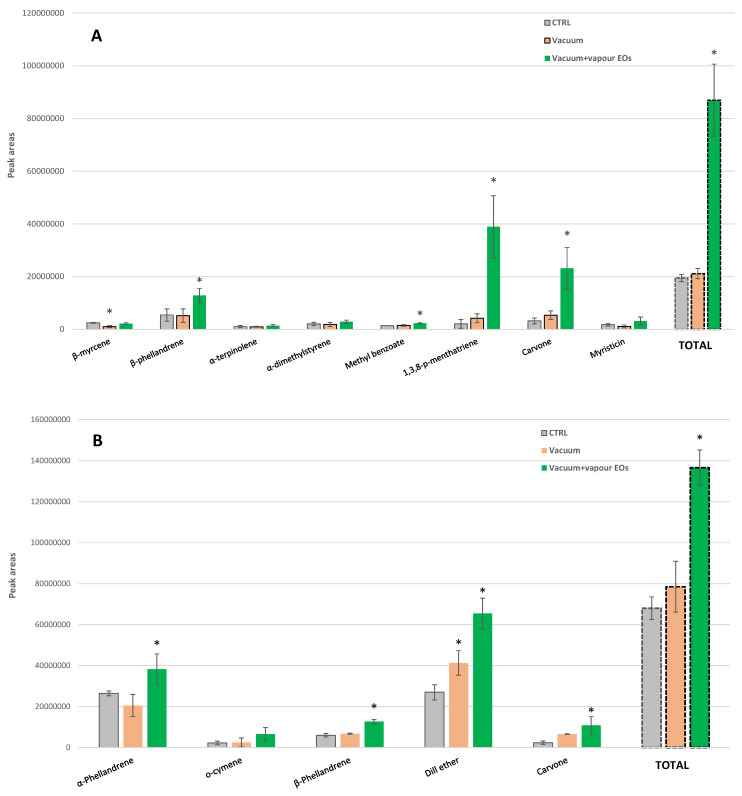
Volatile organic compounds profile of (**A**) curly parsley and (**B**) dill, untreated (CTRL) and treated with vacuum cooling, with and without application of vapor essential oils (EOs), at pilot plant scale (mean ± SD). * Significant differences (*p* < 0.05) in treated samples (either vacuum or vacuum and EOs) compared to CTRL are designated with asterisks.

The effects of the vapor EOs application under vacuum conditions were studied in parsley and dill because of the high commercial importance of these two culinary herbs (Figure 8A,B).

The single vacuum process (without EOs) induced different effects on the individual VOCs of the studied herbs. Thus, *β*-myrcene decreased in parsley by 58% after vacuum treatment (Figure 8A). The same trend was observed in dill for α-phellandrene, although such a reducing trend was not significant (Figure 8B). Meanwhile, dill ether showed the opposite behavior with 53% higher content than the CTRL samples. The rest of the VOCs showed a similar increasing trend after vacuum, although it was not significant. Similarly, an increasing trend was observed for the total VOCs content of samples after vacuum compared to the CTRL samples. This VOCs increment trend could be expected because of cell disruption under vacuum conditions. Then, these burst VOCs release after vacuum treatment will result in their subsequent storage in a product with a lower aroma. The loss of volatile compounds under vacuum treatments has already been observed in liquid products (fruit juices and milk) and bread [15,16,17]. Hence, the VOCs of plant cells might be easily entrained by the evaporated vapor, causing a higher availability of aroma that would lead to an easy aroma loss in the subsequent product storage.

Treatment of curly parsley with vapor EOs under vacuum conditions led to approximately 4.5-fold higher contents of total VOCs in comparison with the CTRL samples (Figure 8A). In particular, *β*-phellandrene content, the major parsley VOC, incremented by 2.4-fold. We observed major increments for 1,3,8-p-menthatriene and carvone with 18.7- and 7.3-fold higher contents, respectively, while methyl benzoate increased by 1.6-fold.

A similar VOCs recovery behavior with EO treatment was observed in dill with a 2-fold higher total VOCs content (Figure 8B). In particular, carvone showed the highest increase of 4.6-fold after vapor EOs compared to the CTRL, while dill ether, *β*-phellandrene and α-phellandrene augmented by 2.4-, 2.1- and 1.4-fold, respectively.

VOCs are spontaneously released by healthy plants, but the released VOC amounts may be enhanced, and even biosynthesis of de-novo VOCs may occur when plants are exposed to stresses (biotic or abiotic) [18,46]. We have observed in our previous studies that EO vapor treatments trigger physiological responses in fruit and vegetables, leading to lower product weight loss, firmness retention and reduction of browning and yellowing, among others [1,13,47]. Indeed, enhancement of antioxidant enzyme systems (superoxide dismutase, catalase, ascorbate peroxidase, etc.) has been observed in several plant products after EO treatments (EO vapor, edible coatings with EOs, etc.) [48,49], which may lead to the consideration of EO treatments by plant cells as abiotic stresses. In addition, we recently found that EOs released from active packaging triggered antioxidant responses in plant cells, with enhanced total phenolic contents and total antioxidant capacity in flat peaches, as well as a reduction of quality-degrading enzymes like polygalacturonase and pectinmethylesterase [10]. Loreto et al. [50] previously reported that monoterpenes content of evergreen oak was increased as a protective mechanism against oxidative stress produced by ozone gas. Furthermore, they demonstrated a common origin and a common functional role of monoterpenes to the well-known antioxidant response of plants to the volatile isoprene. Thus, it was demonstrated that isoprene produced by leaves protected the photosynthetic apparatus against ozone gas stress quenching formed H_2_O_2_ and reduced lipid peroxidation of cellular membranes [51]. Lately, Vickers et al. proposed the “single biochemical mechanism for multiple physiological stressors” model, which showed that volatile isoprenoids may exert protective effects through antioxidant activity [52]. The mechanism by which volatile isoprenoids moderate oxidative loads is still a matter of debate, but the current evidence points to two main avenues: either direct reactions between volatile isoprenoids and oxidizing species and/or mediation of the signaling responses [46,52].

Overall, this study shows how EO vapor treatment under vacuum conditions leads to the recovery aroma profile of aromatic herbs by an antioxidant plant cell response, similar to previous studies with other gas abiotic stresses.

### 3.4. Validation (Industrial Scale) of Aroma Recovery by Vapor Essential Oils Applied under Vacuum Conditions

The observed aroma recovery with vapor EOs under vacuum conditions at the pilot plant scale was validated at the industrial scale with the vacuum cooling system adapted for the SDuVC technology designed by our group.

**Figure 9:**
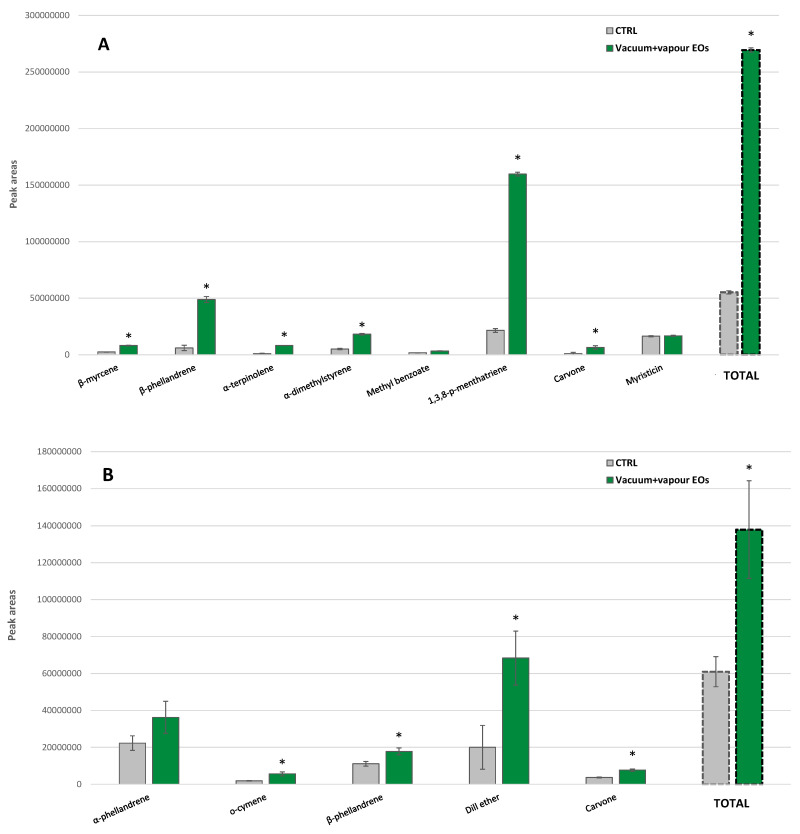
Volatile organic compounds profile of (**A**) curly parsley and (**B**) dill, untreated (CTRL) or after vacuum cooling and vapor essential oils (EOs) treatment, at the industrial scale (validation) (mean ± SD). * Significant differences among the EOs and CTRL samples are denoted by an asterisk at *p* < 0.05 according to an independent samples *t*-test.

The parsley aroma related to the total VOCs content was recovered by 4.9-fold after the vacuum and EO treatment (Figure 9A). Regarding individual VOCs, *α*-terpinolene, *β*-phellandrene, 1,3,8-p-menthatriene, carvone, *β*-myrcene and α-dimethylstyrene were incremented by 8.8-, 8.1-, 7.4-, 6.2-, 3.3- and 3.6-fold, respectively, after the vacuum and EO treatment, which were slightly higher than the increments observed at the pilot plant scale.

The dill aroma also was recovered by 2.3-fold (total VOCs) after the vacuum and EO treatment (Figure 9B). Dill ether, o-cymene and carvone were increased by 3.4-, 3.0- and 2.1-fold, respectively, while β-phellandrene was augmented by 1.6-fold after the vacuum and EO treatment.

As observed, the aroma recovery at the pilot plant scale was also obtained at the industrial scale with the vacuum cooling system adapted for the SDuVC technology. Furthermore, such aroma recovery was higher at the industrial scale, which may be explained by the longer treatment time of the industrial vacuum cooling process (15 min is the total time of vacuum cooling process and EO vapor treatment). The relative content (%) of the individual VOCs to the total VOCs profile (100%) also varied after the vacuum cooling process and EO vapor treatment. Nevertheless, the major VOCs characteristic of curly parsley and dill were always enhanced. Samples treated with the vacuum and EO vapor treatment were more aromatic, as scored in the informal panel tests conducted after this treatment in the industrial installations. Nevertheless, future studies with trained sensory panel tests must be conducted to correlate such observed higher VOCs contents with sensory scores.

The aroma recovery of plant products, and aromatic herbs in particular, by applying vaporized EOs under vacuum conditions is hereby proposed to compensate for the reduction of VOCs levels after postharvest treatments such as vacuum cooling. Hence, product aromatic quality is better maintained, leading to longer product shelf life and lower food waste. This aspect is of high interest since a shrink rate in celery of 8.5% was estimated in a study of US supermarkets in 2011–2012 [53]. Nevertheless, such waste ratios may dramatically increase in other culinary herbs with higher postharvest respiration and ethylene production rates (e.g., dill, parsley, etc.), or in those that are very sensitive to chilling injury, like basil [3].

## 4. Conclusions

The aroma of culinary herbs is defined by their volatile organic compounds profile, which must be preserved during their postharvest life using different postharvest technologies. This study shows how the application of the innovative technology of essential oils vaporized during the vacuum cooling process of culinary herbs enhances their aroma profile to compensate for subsequent aroma losses that may occur during the herbs’ shelf life. In particular, the aroma quality of curly parsley and dill was recovered at both the pilot plant scale and the industrial level (validation). Furthermore, solid-phase microextraction (SPME) sorption of the volatile organic compounds was modeled to better characterize the sorption equilibrium between the analyte and the SPME stir-bar coating. Hence, this technology is proposed to be combined with vacuum cooling devices installed in culinary herbs processing plants. 

## Figures and Tables

**Table 1:** Spearmint essential oil composition.

Compound	%
Carvone	81.4
Limonene	4.78
cis-dihydrocarvone	2.30
trans-dihydrocarvyl acetate	1.93
Menthone	0.99
cis-carvyl acetate	0.34
*β*-bourbonene	0.12
Octanol-3	0.11
Sabinene hydrate	0.02
cis-jasmone	0.01
Viridiflorol	0.01

**Table 2:** Headspace solid-phase microextraction sorption model parameters.

	Specific Sorption Rate (min^−1^)	C_max_^1^ (ln Peak Area)	RMSE ^2^ (ln Peak Area)
**Monoterpene hydrocarbons**			
*α*-pinene	0.0063 ± 0.0020	2.76 ± 0.045	0.070
Sabinene	0.0042 ± 0.0009	3.17 ± 0.037	0.047
*β*-pinene	0.0052 ± 0.0020	2.91 ± 0.029	0.025
*β*-myrcene	0.0223 ± 0.0032	3.31 ± 0.009	0.036
3-carene	0.0229 ± 0.0056	3.09 ± 0.014	0.038
α-terpinene	0.0420 ± 0.0067	3.09 ± 0.006	0.025
Limonene	0.0174 ± 0.0041	3.10 ± 0.008	0.025
trans-ocimene	0.0168 ± 0.0026	3.49 ± 0.010	0.033
*γ*-terpinene	0.0115 ± 0.0016	3.17 ± 0.009	0.027
**Oxygenated monoterpenes**			
1,8-cineole	0.0296 ± 0.0027	3.74 ± 0.005	0.016
4-thujanol	0.0156 ± 0.0017	3.11 ± 0.008	0.023
α-terpinolene	0.0134 ± 0.0016	3.24 ± 0.010	0.031
Linalool	0.0079 ± 0.0011	3.79 ± 0.010	0.025
Camphor	0.0103 ± 0.0024	3.23 ± 0.012	0.034
Borneol	0.0119 ± 0.0018	2.77 ± 0.009	0.021
4-terpineol	0.0085 ± 0.0009	3.34 ± 0.011	0.027
α-terpineol	0.0098 ± 0.0016	3.11 ± 0.012	0.033
Bornyl acetate	0.0137 ± 0.0026	3.1 ± 0.0076	0.020
**Sesquiterpene hydrocarbons**			
Bicycloelemene	0.0130 ± 0.0019	3.05 ± 0.016	0.044
*α*-copaene	0.0120 ± 0.0018	3.16 ± 0.011	0.033
*α*-cubebene	0.0115 ± 0.0013	3.01 ± 0.009	0.030
*β*-elemene	0.0138 ± 0.0016	3.43 ± 0.010	0.035
*β*-caryophyllene	0.0132 ± 0.0015	3.24 ± 0.010	0.031
*β*-cubebene	0.0145 ± 0.0018	3.01 ± 0.009	0.031
*α*-guaiene	0.0123 ± 0.0017	3.25 ± 0.009	0.032
*β*-bergamotene	0.0186 ± 0.0020	3.01 ± 0.007	0.028
*β*-farnesene	0.0179 ± 0.0019	3.59 ± 0.008	0.030
epi-bicyclo sesquiphellandrene	0.0152 ± 0.0023	3.12 ± 0.013	0.064
*γ*-muurolene	0.0156 ± 0.0019	2.93 ± 0.008	0.030
Germacrene D	0.0192 ± 0.0026	3.58 ± 0.010	0.039
*α*-bulnesene	0.0133 ± 0.0017	3.45 ± 0.013	0.038
*δ*-guaiene	0.0151 ± 0.0017	3.39 ± 0.001	0.034
*γ*-cadinene	0.0144 ± 0.0016	3.43 ± 0.001	0.035
Sesquisabinene	0.0181 ± 0.0020	3.15 ± 0.001	0.036
**Oxygenated sesquiterpenes**			
T-cadinol	0.0187 ± 0.0021	3.09 ± 0.013	0.042
**Aromatic compounds**			
Cis methyl cinnamate	0.0095 ± 0.0012	3.36 ± 0.008	0.021
Eugenol	0.0260 ± 0.0052	3.48 ± 0.010	0.037
trans methyl cinnamate	0.0191 ± 0.0035	3.78 ± 0.006	0.023
Methyleugenol	0.0206 ± 0.0022	3.52 ± 0.007	0.026

^1^*C_max_*, maximum peak area (equilibrium); ^2^ RMSE, root-mean-squared error.

## Data Availability

The data presented in this study are available on request from the corresponding author.
